# Associations Between Household Chaos and Appetitive Traits in Preschoolers and Preadolescents

**DOI:** 10.21203/rs.3.rs-6567466/v1

**Published:** 2025-12-16

**Authors:** Zhuoya Zhang, Delaina Carlson, Caroline Borowy, Mingliang Ge, Diane Gilbert-Diamond, Jennifer A. Emond

**Affiliations:** Dartmouth College; Dartmouth College; Dartmouth College; Dartmouth College; Dartmouth College; Dartmouth College

**Keywords:** household chaos, appetitive trait, childhood obesity, hedonic eating, preschooler, preadolescent, middle childhood, eating behavior, eating in the absence of hunger

## Abstract

**Background:**

Previous research suggests household chaos may relate to greater childhood obesity risk, though mechanisms are unclear. This study aimed to evaluate the cross-sectional associations between household chaos and appetitive traits in 92 preschoolers (Study A) and 184 preadolescents (Study B).

**Methods:**

Parents completed the Confusion, Hubbub, and Order Scale and the Child Eating Behavior Questionnaire. Eating in the absence of hunger (EAH) was assessed in the lab. Data were collected at baseline and either a six-month (Study A) or one-year (Study B) follow-up. Intra-class correlation coefficients (ICC) were computed to assess the stability of household chaos and appetitive traits. Associations of household chaos with appetitive traits were examined via linear mixed-effect models, adjusting for child age, sex, and annual household income. Sensitivity analyses further adjusted for child nighttime sleep duration, number of children in the household, and parenting style.

**Results:**

Household chaos (preschoolers: 6-month ICC = 0.93; preadolescents: 1-year ICC = 0.85) and child appetitive traits (preschoolers ICC: 0.56–0.77; preadolescents ICC: 0.51–0.74) showed moderate to high stability. In both cohorts, chaos was positively associated with emotional overeating (preschoolers: standardized beta coefficient β_s= 0.18, 95% CI: 0.00, 0.35; preadolescents: β_s= 0.19, 95% CI: 0.07, 0.31). In preadolescents, more household chaos was related to higher food responsiveness (β_s= 0.21, 95% CI: 0.09, 0.32) and lower satiety responsiveness (SR, β_s = −0.14, 95% CI: −0.25, −0.02). Chaos was positively related to SR in preschoolers (β_s = 0.20, 95% CI: 0.03, 0.37); however, this association became statistically non-significant after adjusting for sleep. No associations were found for enjoyment of food, emotional undereating, and EAH.

**Conclusions:**

Our findings suggest that household chaos may contribute to childhood obesity by affecting children’s appetitive traits.

## 1. Background

Appetitive traits, defined as predispositions to approach or avoid food (1), can contribute to childhood obesity. A meta-analysis of epidemiologic studies in children aged 1 month to 13 years found that food approach appetitive traits, such as food responsiveness (FR, i.e., strong desire for food, *r* = 0.22, 95% CI: 0.16, 0.29) and emotional overeating (EOE, *r* = 0.15, 95% CI: 0.08, 0.22) are positively related to child weight status (2). Similarly, eating in the absence of hunger (EAH), a lab-observed measure of responsivity to food and food cues (1), is positively linked to weight status in children aged 12 years and under (3). In contrast, food avoidance appetitive traits are theorized to protect against childhood overweight and obesity. For instance, satiety responsiveness, i.e., restraint from eating when satiated, is related to lower child weight status (*r* = −0.21, 95% CI: −0.24, −0.17) (2). In adolescents aged 10 to 12 years, higher FR and EOE and lower SR were further associated with higher cardiometabolic risk (4), suggesting that these appetitive traits may negatively impact child cardiometabolic health. Notably, longitudinal studies indicated moderate to high stability of these appetitive traits from age 4 to 10 (*r* = 0.44–0.55) and from age 7 to 13 (intra-class correlations, ICC’s: 0.63–0.83) (5). Therefore, appetitive traits can persist in childhood and influence children’s long-term health (6).

According to the Family Ecological model, the family context shapes the development of child health behaviors (7). Organization within the household can offer children structure and routines in daily activities, such as consistent mealtime and sleep schedules (8, 9). A structured family environment can reduce the child’s stress around these activities and encourage positive parent-child interactions (10). In contrast, disorganization and chaos within the household can make it difficult for children to engage in regular healthful behaviors (11). Household chaos is characterized by excessive instability (i.e., hurriedness and lack of family routines) and disorganization (i.e., high background noise, crowding, and clutter) (12). Studies have shown that children living in more chaotic family environments experienced poorer sleep quality (13) and more sleep problems (14–16). Further, a cross-sectional study in preschoolers showed that household chaos was associated with higher total media use and media use related to sleep disruption (17). Several studies found that household chaos was positively related to weight status in infants (18) and preschoolers (19).

However, limited research has explored the link between chaos and child eating behaviors. Two studies examined this question in young children, suggesting that household chaos is related to more parent-reported emotional overeating (19, 20). Yet, these findings have not been confirmed with lab-observed measures of hedonic eating. Further, how household chaos would affect appetitive traits in middle childhood remains unexplored. Importantly, structure and orderliness within the family are crucial to this developmental stage, which offers the security and emotional stability needed to navigate the physical and emotional changes from childhood to adolescence (22). Understanding the impact of household chaos on child appetitive traits can inform efficacious childhood obesity interventions to target modifiable aspects of the household environment.

This study aims to evaluate the associations between household chaos and appetitive traits (parent-reported and lab-observed) in independent samples of preschool-aged children and preadolescents. We hypothesized that in both groups, (1) household chaos would be positively associated with food responsiveness, emotional overeating, enjoyment of food, and EAH, and (2) household chaos would be negatively associated with satiety responsiveness and emotional undereating.

## 2. Methods

### 2.1. Study Samples

This study is a secondary analysis of data collected from two independent cohorts. Data include (1) parent responses to questionnaires and (2) lab-observed eating in the absence of hunger. Study A collected data at baseline (N = 92) and a six-month follow-up (N = 80). Study B collected data at baseline (N = 184) and a one-year follow-up (N = 95). Participants who completed the follow-up visits had similar demographics as those who completed the baseline visit (*p* > 0.05, Table S1).

Study A enrolled children aged 3 to 5 years and a parent (including guardians) via flyers and email listservs from New Hampshire and Vermont from July 2018 through October 2022. The study evaluated the impact of media use on young children’s sleep and eating behaviors. Children were eligible if they could comprehend English; had no significant developmental or language delay, sleep disorders, food allergies or dietary restrictions, appetite- or attention-altering medications or disorders; had no relatives in this study; no plan to relocate in six months; and lived with the enrolled parent for more than half the time. Parents provided written consent, and children provided verbal assent at the baseline.

Study B enrolled children aged 9 to 12 years and a parent (including guardians) from the same geographical areas as Study A from April 2018 through April 2023. The original study examined the association between food-cue-related neural reward reactivity and EAH behavior and potential effect modification by obesity-related genotypes. Children were eligible if they could comprehend English; had no relevant food allergies or dietary restrictions; did not take/have appetite- or attention-altering medications or disorders, psychiatric and neurological disorders; had no relatives in the study; and were willing to participate in all study visits. Because this study involved magnetic resonance imaging, children were excluded if they had metal in or on the body or claustrophobia. Parents provided informed written consent, and children provided informed written assent at the baseline.

Dartmouth College’s Committee for the Protection of Human Subjects approved the study protocols (Study A: CPHS31097; Study B: CPHS30723).

### 2.2. Exposure Variable: Household Chaos

Parents completed the Confusion, Hubbub, and Order Scale (CHAOS), a validated questionnaire on household chaos (23). Parents rated their own household on 15 statements describing characteristics of a chaotic household environment, e.g., “No matter how hard we try, we always seem to be running late” and “Our home is a good place to relax” (reverse scored). Responses range from 1 (very much like your own home) to 4 (not at all like your own home). Total scores were the sum across items, ranging from 15 to 60. Higher scores indicate a more chaotic household. The scale showed acceptable internal consistency in the original study (Cronbach’s *α* = 0.79) and in our samples (Study A: *α* = 0.85; Study B: *α* = 0.87) (23). The scale showed high one-year test-retest reliability (*r* = 0.74) and convergent validity against observed measures of household organization in the original study (23). While unrelated to socioeconomic status (SES) in the original study (23), the scale correlated with annual household income, number of children in the household, and home ownership status in another study among preschoolers (17).

### 2.3. Outcome Variables: Appetitive Traits

Parents completed the Child Eating Behavior Questionnaire (CEBQ) to assess children’s appetitive traits (24). Our analysis considered three subscales on food approach traits: food responsiveness (FR, five items, e.g., “If allowed to, my child would eat too much.”), emotional overeating (EOE, four items, e.g., “My child eats more when worried.”), and enjoyment of food (EF, four items, e.g., “My child loves food.”). Two food avoidance traits were also assessed: satiety responsiveness (SR, five items, “e.g., “My child gets full before his/her meal is finished.”) and emotional undereating (EUE, four items, e.g., “My child eats less when angry.”). Parents rated each item on a five-point Likert scale from 1 (never) to 5 (always). Subscale scores were the average responses across items (range: 1 to 5). Higher scores indicate more parent-perceived presence of the appetitive traits. Subscales showed acceptable to high internal consistency in our samples (Study A: *α* range: 0.69–0.91; Study B: 0.77–0.83) (Table S2).

In both studies, children completed the eating in the absence of hunger experiment in our lab at baseline and follow-up visits (3). After having a preload meal, children were provided with snacks in open bowls to eat ad-libitum (Study A: 60 g ± 5 g of Goldfish crackers, 60 g ± 5 g of mini graham crackers; Study B: 240 g ± 1 g of gummy bears, 76 g ± 0.5 g of Goldfish crackers, 235 g ± 1 g of grapes, and water) while watching a TV show embedded with food commercials. Trained research staff weighed the snacks before and after the EAH experiment. EAH was operationalized as the caloric intake during the experiment, computed by multiplying each EAH snack’s known energy content per gram by the amount consumed.

### 2.4. Covariates

#### 2.4.1. Demographics

In both studies, parents reported children’s date of birth, sex, race, and ethnicity in the baseline questionnaire. Parents reported annual household income at baseline. In the Study A survey, parents also reported the number of children living in the household at baseline.

#### 2.4.2. Parenting Style

In Study A, parents rated their parenting style with the Parenting Style and Dimensions Questionnaire-short form (PSDQ) at baseline (25). Study A considered permissive parenting (five items, e.g., “I find it difficult to discipline my child,” *α* = 0.68) and authoritative parenting subscale (15 items, e.g., “I give my child reasons why rules should be obeyed,” *α* = 0.85) from the PSDQ. Subscale scores range from 1 to 5, where higher scores indicate a more authoritative or permissive parenting style. In Study B, children assessed their parents’ parenting style with the Authoritative Parenting Index (API) at baseline (26). The API has two subscales. Demandingness was assessed with four items, e.g., “She/he has rules that I must follow,” with subscale scores ranging from 4 to 16 (*α* = 0.52). Responsiveness was evaluated with five items, e.g., “She/he listens to what I have to say,” with subscale scores ranging from 5 to 20 (*α* = 0.75). Higher scores reflect more responsive or demanding parenting.

#### 2.4.3. Nighttime Sleep Duration

Actigraphy was used to collect children’s sleep patterns in the home environment at baseline and follow-up visits in both studies. Raw accelerometry data was processed separately for each cohort due to the age difference. Study A participants wore ActiGraph wGT3X-BT (Pensacola, FL) on their non-dominant ankles, day and night, for at least five consecutive days. Parents reported their child’s bedtime and wake time on a sleep log. The Sitnik algorithm was used to detect waketime if it was missing from the sleep log (27). Parents also recorded any non-wear periods, which were excluded from the analysis. The nighttime sleep period was identified as the period from parent-reported bedtime to wake time the next morning. Within that window, each minute was classified as sleep or wake with the Sadeh algorithm (28, 29). Study B participants wore Actigraph GT9X Link on their non-dominant wrists for at least five days. Raw accelerometry data was processed into 60-second epochs on the ActiLife 6 software (30). Each minute was marked as sleep or wake with the Cole-Kripke algorithm (31). Sleep periods (from bedtime to waketime the next morning) were identified using the Tudor-Locke algorithm (32). Non-wear periods were detected with the Troiano algorithm and were excluded from the analysis (33).

Nighttime sleep duration was computed by summing the minutes marked as asleep for each overnight sleep period. In both studies, participants with at least three nights of valid data were included in the analysis (Study A: baseline N = 73, month 6: N = 50; Study B: baseline N = 154, one-year: N = 79). The average nighttime sleep duration was computed using the mean sleep duration across all valid nights.

#### 2.4.4. Anthropometry

Trained research staff measured participant weight and height three times during in-person visits. Study A collected anthropometry at baseline only, because in-person visits were paused during the COVID-19 pandemic (N = 52). Study B collected anthropometry at baseline (N = 183) and one-year follow-up (N = 94). Age and sex-standardized body mass index z-scores (BMI-z) were computed using the 2000 CDC growth charts (34).

### 2.5. Statistical Analysis

First, descriptive statistics were computed for all variables to summarize participant characteristics at baseline. Next, Pearson’s Chi-squared tests and Welch’s two-sample t-tests were used to compare the distribution of sociodemographic characteristics between participants in the baseline samples and those who computed the follow-up visits. Additionally, bivariate analysis between household chaos and participant baseline demographics was performed via the Spearman correlation test, Wilcoxon rank sum test, or Jonckheere-Terpstra test for trend, as appropriate. Linear mixed-effect models with random intercepts were fit to estimate the association between household chaos and each appetitive trait, accounting for the repeated measures per child. Child age, sex, and annual household income were included in all models.

As sensitivity analyses, models were further adjusted for parenting style, child nighttime sleep duration, and the number of children in the household to examine the robustness of the observed associations. A directed acyclic graph was created to identify potential confounders and mediators (Figure S1) (35). Further, potential effect modification by child sex was examined by including an interaction term between chaos and the corresponding appetitive trait in the model. Additionally, the association between household chaos and child BMI-z was explored via linear regression or linear mixed-effect models, as appropriate. The intra-class correlation coefficient (ICC, two-way random effect model) was computed for household chaos and appetitive traits to evaluate the stability of these constructs over time. Based on past research, an ICC between 0.50 and 0.75 was considered moderate, 0.75 and 0.90 good, and above 0.90 excellent reliability (36). All statistical analysis was conducted with R version 4.3.2 (R Core Team, 2023). A *p*-value < 0.05 was considered as statistical significance.

## 3. Results

### 3.1. Participant Characteristics

In Study A, participants were, on average, 4.2 years old (SD = 1.0), with 44% female at baseline (Table 1). In Study B, the average participant baseline age was 10.9 (SD = 1.2), with 42% female. In both samples, most participants were white and non-Hispanic (Study A: 88%; Study B: 90%). In both samples, most children were from households with annual incomes above $65,000 (Study A: 72%; Study B: 87%). Twenty-three percent of preschoolers and 31% of preadolescents were with overweight or obesity.

The average score on the CHAOS scale was 28.2 (SD = 6.6) in Study A and 28.5 (SD = 7.1) in Study B. In Study A, families with more children living in the household scored higher on the CHAOS scale (*p* < 0.001, Table S3). In Study B, lower household income was related to more household chaos (*p* = 0.003, Table S4).

### 3.2. Stability of Household Chaos and Appetitive Traits

Household chaos presented high stability in Study A (six-month ICC = 0.93) and Study B (one-year ICC = 0.85) (Table S5). Additionally, parent-reported appetitive traits showed moderate to high stability with six-month ICCs ranging from 0.56 (EUE) to 0.77 (FR) in preschoolers and stability of one-year ICCs ranging from 0.59 (EOE and EUE) to 0.74 (FR) in preadolescents (Table S5).

EOE showed a statically significant time effect in preschoolers (*p* = 0.009), where participants scored, on average, 0.14 points (95% CI: 0.04, 0.25) higher on EOE at the six-month follow-up than baseline. A positive time trend was also found for EAH in preadolescents (*p* = 0.009). The calories consumed during the EAH experiment at the one-year follow-up visit was, on average, 56.07 kcal (95% CI: 13.99, 98.16) higher than baseline. No statistically significant main time effect was observed for other appetitive traits.

### 3.3. Associations Between Household Chaos and Appetitive Traits

**Figure 1:**
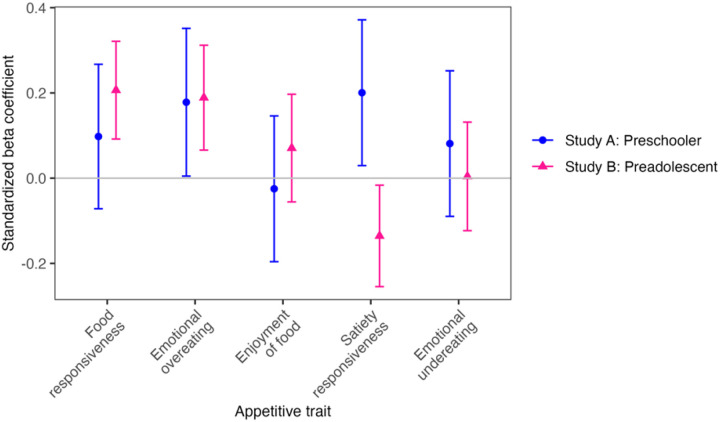
Associations between household chaos and appetitive traits in preschoolers and preadolescents. *Notes*: Standardized beta coefficients were derived from linear mixed-effect models with household chaos as the independent variable and each appetitive trait as the dependent variable. Models were adjusted for child age, sex and annual household income.

Household chaos was positively related to EOE in preschoolers (standardized beta coefficient, *β*_*s*_ = 0.18, 95% CI: 0.00, 0.35, *p* = 0.04) and preadolescents (*β*_*s*_ = 0.19, 95% CI: 0.07, 0.31) (Fig. 1, Table S6). Additionally, chaos was positively related to FR in preadolescents (*β*_*s*_ = 0.21, 95% CI: 0.09, 0.32) (Fig. 1, Table S6).

The association for satiety responsiveness differed by age group (Fig. 1, Table S6). Household chaos was positively related to SR in preschoolers (*β*_*s*_ = 0.20, 95% CI: 0.03, 0.37) but inversely associated with SR in preadolescents (*β*_*s*_ = −0.14, 95% CI: −0.25, −0.02). No associations were found with EF, EUE, EAH, or BMI-z in either group (Table S6).

### 3.4. Sensitivity Analysis

In preschoolers, adjusting for the number of children in the household attenuated the association between chaos and EOE towards null (Table S7). Further, the association between chaos and SR became statistically non-significant after adjudging for nighttime sleep duration in preschoolers (Table S7). The observed associations in preadolescents remained similar after further adjusting for parenting style and sleep duration (Table S8). No significant effect modification by sex was observed (*p*-values for the interaction terms > 0.10).

## 4. Discussion

This study examined the cross-sectional associations between household chaos and appetitive traits in two cohorts of children in early and middle childhood from samples primarily consisting of families from a higher SES. In summary, household chaos was related to appetitive traits that have been associated with greater obesity risk in both groups. Findings suggest household chaos may contribute to childhood obesity by impacting appetitive traits.

In both groups, household chaos was positively related to EOE. This finding aligns with earlier research, which reported a positive association between household chaos and EOE in toddlers (*β*_*s*_ = 0.12, *p* = 0.05) (21) and in preschoolers (*r* = 0.20, *p* < 0.05) (20). While our observation in Study A confirmed prior research, our findings among preadolescents suggest that this chaos-EOE association is not specific to young children. Increased EOE may result from heightened stress from living in a chaotic home (20). Evidence shows that high levels of household chaos can increase child stress and negative affect (37, 38). Further, in children aged 6 to 9 years, Hong and colleagues found that more household chaos was related to lower child emotion regulation (39). Possibly, emotional overeating may serve as a maladaptive coping mechanism for children to regulate negative affect (i.e., frustration, fear, and anger) due to constant chaos within the family (40–42). Mediation analysis should explore the underlying pathways between household chaos and child emotional overeating. Interestingly, our sensitivity analysis showed that adjusting for the number of children in the household attenuated the chaos-EOE association towards null in preschoolers. The number of children in the household positively correlated with both household chaos (*r* = 0.51, *p* < 0.0001 via a Spearman’s correlation test) and EOE (*r* = 0.28, *p* = 0.007) in Study A. We were not able to replicate this analysis in the preadolescent sample, because family structure was not assessed in Study B. Further research should explore the potential confounding of family structure on the chaos-EOE association.

In preadolescents, we observed that household chaos was related to increased FR and reduced SR (Fig. 1). Lumeng and colleagues reported similar associations for FR (*r* = 0.21, *p* < 0.001) in preschool-aged children of lower SES (20). Potentially, the lack of daily activity routines at home can interrupt children’s circadian rhythm and appetite regulation, which can increase their reactivity to food cues (43, 44). In preschoolers, we found that household chaos was related to more SR in the main model (Fig. 1). This finding was unexpected, because household chaos was hypothesized to increase distractions and arousal for children, reducing their awareness of internal satiety cues (45). However, after adjusting for nighttime sleep duration, the chaos-SR association became statistically non-significant (Table S7). This attenuation may result from a lower statistical power, as some participants did not have sleep data. It is also possible that poor sleep quality mediates the association between chaos and reduced satiety responsiveness. Studies in children showed that household chaos was related to more sleep problems (14–16) and shorter sleep (46), both of which are related to lower SR (47, 48). Longitudinal research should investigate whether sleep duration, quality, and regularity mediate the association between household chaos and satiety responsiveness in children.

This study has strengths and limitations. First, our study samples included children at two distinct developmental stages: early and middle childhood. This study extended the literature on the associations between household chaos and appetitive traits in older children (49). Second, repeated measurements allowed us to assess the stability of household chaos and child appetitive traits and increased the power of our statistical analysis. The study limitations include the homogeneity of participant socioeconomic profiles and potential unmeasured confounding. Further, household chaos and appetitive traits were assessed via questionnaires from the same parent, which may increase response bias (50). Future studies should consider collecting data from multiple informants. The cross-sectional study design limited causal inferences. Lastly, children in both samples were primarily White, non-Hispanic, and of higher SES, which may limit the study’s generalizability. Future research should confirm our findings in longitudinal studies with larger, more racially and socio-economically diverse samples.

## 5. Conclusions

In preschoolers and preadolescents, household chaos was positively related to emotional overeating. Additionally, more chaos at home was associated with reduced satiety responsiveness and increased food responsiveness in preadolescents. These findings suggest that household chaos may contribute to childhood obesity by affecting appetitive traits.

## Supplementary Files

This is a list of supplementary files associated with this preprint. Click to download.

- BMCChaosATSupplementaryMaterial.pdf

## Figures and Tables

**Table 1:** Participant baseline characteristics.

	Study A: Preschoolers	Study B: Preadolescents
	N = 92	N = 184
	Mean ± SD or N (%)	Mean ± SD or N (%)
Child age (years)	4.2 ± 1.0	10.9 ± 1.2
Female	40 (44%)	77 (42%)
White non-Hispanic	81 (88%)	166 (90%)
With overweight or obesity^a^	12 (23%)	57 (31%)
Annual household income ($)^b^		
<65,000	26 (28%)	24 (13%)
65,000–145,000	47 (51%)	99 (54%)
145,000–225,000	13 (14%)	43 (23%)
>225,000	6 (7%)	18 (10%)
Household chaos (CHAOS)^c^	28.2 ± 6.6	28.5 ± 7.1

a Weight and height were collected during the baseline in-person visit for 52 children in Study A and 183 children in Study B.

b Household income was missing for 4 Study A participants and was imputed with the sample mode.

c Household chaos was assessed with the Confusion, Hubbub, and Order Scale (CHAOS, range: 15 to 60).

**Table 2:** Distribution of baseline child appetitive traits and weight status.

	Study A: Preschoolers		Study B: Preadolescents	
	N	Mean ± SD	N	Mean ± SD
Food responsiveness^a^	92	2.35 ± 0.70	184	2.58 ± 0.74
Emotional overeating	92	1.56 ± 0.53	184	2.21 ± 0.66
Enjoyment of food	92	3.70 ± 0.80	184	3.94 ± 0.59
Satiety responsiveness	92	3.22 ± 0.71	184	2.76 ± 0.64
Emotional undereating	92	2.54 ± 0.72	184	2.93 ± 0.72
EAH (kcal)^b^	52	79.17 ± 71.47	184	439.97 ± 222.06
BMI-z^c^	52	0.40 ± 1.06	183	0.48 ± 1.00

a Child appetitive traits (parent-report) were assessed with subscales from the Child Eating Behavior Questionnaire (range: 1 to 5).

b EAH, eating in the absence of hunger was operationalized as the caloric intake during the EAH experiment.

c Age- and sex-adjusted body mass index z-scores.

## Data Availability

The datasets used and analyzed in the current study are available from the corresponding author upon reasonable request.

## Abbreviations

FR: food responsiveness
EOE: emotional overeating
EF: enjoyment of food
SR: satiety responsiveness
EUE: emotional undereating
BMI-z: age- and sex-adjusted body mass index z-scores
EAH: eating in the absence of hunger
SES: socio-economic status
CI: confidence interval
CHAOS: Confusion, Hubbub, and Order Scale
CEBQ: Child Eating Behavior Questionnaire
PSDQ: Parenting Style and Dimensions Questionnaire-short form
API: Authoritative Parenting Index
ICC: intra-class correlation
